# Interplay of choline metabolites and genes in patient-derived breast cancer xenografts

**DOI:** 10.1186/bcr3597

**Published:** 2014-01-21

**Authors:** Maria T Grinde, Nirma Skrbo, Siver A Moestue, Einar A Rødland, Eldrid Borgan, Alexandr Kristian, Beathe Sitter, Tone F Bathen, Anne-Lise Børresen-Dale, Gunhild M Mælandsmo, Olav Engebraaten, Therese Sørlie, Elisabetta Marangoni, Ingrid S Gribbestad

**Affiliations:** 1 Department of Circulation and Medical Imaging, NTNU, Trondheim, Norway; 2 St. Olavs University Hospital, Trondheim, Norway; 3 Department of Tumor Biology, Oslo University Hospital, Norwegian Radium Hospital, Oslo, Norway; 4 Department of Genetics, Institute for Cancer Research, Oslo University Hospital, Norwegian Radium Hospital, Oslo, Norway; 5 Institute of Clinical Medicine, Faculty of Medicine, University of Oslo, Oslo, Norway; 6 Center for Cancer Biomedicine & Department of Informatics, University of Oslo, Oslo, Norway; 7 K.G. Jebsen Center for breast cancer research, Institute of Clinical Medicine, Faculty of Medicine, University of Oslo, Oslo, Norway; 8 Department of Technology, Sør-Trøndelag University College, Trondheim, Norway; 9 Department of Pharmacy, Faculty of Health Sciences, University of Tromsø, Tromsø, Norway; 10 Cancer Stem Cell Innovation Center, Oslo University Hospital, Norwegian Radium Hospital, Oslo, Norway; 11 Department of Oncology, Oslo University Hospital, Oslo, Norway; 12 Preclinical Investigation Unit, Translational Research Department, Institut Curie, Paris, France

## Abstract

**Introduction:**

Dysregulated choline metabolism is a well-known feature of breast cancer, but the underlying mechanisms are not fully understood. In this study, the metabolomic and transcriptomic characteristics of a large panel of human breast cancer xenograft models were mapped, with focus on choline metabolism.

**Methods:**

Tumor specimens from 34 patient-derived xenograft models were collected and divided in two. One part was examined using high-resolution magic angle spinning (HR-MAS) MR spectroscopy while another part was analyzed using gene expression microarrays. Expression data of genes encoding proteins in the choline metabolism pathway were analyzed and correlated to the levels of choline (Cho), phosphocholine (PCho) and glycerophosphocholine (GPC) using Pearson’s correlation analysis. For comparison purposes, metabolic and gene expression data were collected from human breast tumors belonging to corresponding molecular subgroups.

**Results:**

Most of the xenograft models were classified as basal-like (N = 19) or luminal B (N = 7). These two subgroups showed significantly different choline metabolic and gene expression profiles. The luminal B xenografts were characterized by a high PCho/GPC ratio while the basal-like xenografts were characterized by highly variable PCho/GPC ratio. Also, Cho, PCho and GPC levels were correlated to expression of several genes encoding proteins in the choline metabolism pathway, including choline kinase alpha (*CHKA)* and glycerophosphodiester phosphodiesterase domain containing 5 (*GDPD5)*. These characteristics were similar to those found in human tumor samples.

**Conclusion:**

The higher PCho/GPC ratio found in luminal B compared with most basal-like breast cancer xenograft models and human tissue samples do not correspond to results observed from *in vitro* studies. It is likely that microenvironmental factors play a role in the *in vivo* regulation of choline metabolism. Cho, PCho and GPC were correlated to different choline pathway-encoding genes in luminal B compared with basal-like xenografts, suggesting that regulation of choline metabolism may vary between different breast cancer subgroups. The concordance between the metabolic and gene expression profiles from xenograft models with breast cancer tissue samples from patients indicates that these xenografts are representative models of human breast cancer and represent relevant models to study tumor metabolism *in vivo*.

## Introduction

Breast cancer is not a single disease with variable morphologic features, but rather a group of molecularly distinct neoplastic disorders [1]. According to the gene expression-based intrinsic classification, breast carcinomas can be categorized into at least five subtypes: luminal A, luminal B, normal breast-like, human epidermal growth factor receptor 2 (HER2) enriched, and a basal-like subtype [2,3]. In addition to the distinctly different gene expression patterns, the subgroups also show significantly different clinical outcomes [3], likely to be caused by alterations in specific cellular pathways. Moreover, tumors that appear to have similar diagnostic features, do not always respond to treatment in the same way. This can, among other factors, be caused by differences in mutational profile, signaling redundancy and the particular tumor microenvironment [4].

Most of the existing *in vivo* preclinical breast cancer models are established from a limited number of cell lines isolated from human tumors grown in cell culture before implantation into immunodeficient animals. These models do not reflect the breast cancer heterogeneity since they usually have a monomorphic, poorly differentiated histology and lack of tissue organization [5]. A panel of patient-derived xenograft models has been established in which human breast tumor tissue has been engrafted directly into mice [6-8]. Patient-derived xenograft models generally maintain key features of the original tumors, including histologic subtype, degree of differentiation, growth pattern, and gene expression profiles, even after several passages *in vivo*[5-10]. Furthermore, the drug response in these models shows a good correlation with the primary patient tumors [5,6,11], and altogether the xenografts are representative model systems for studies of metabolic and genetic patterns in human breast cancer.

Abnormal choline metabolism is a well-known feature of breast cancer. An elevated total choline (tCho) signal can be observed using magnetic resonance spectroscopy (MRS) and is an *in vivo* biomarker for malignant disease [12]. In line with this, a reduction in tCho has been suggested as an *in vivo* marker for response to treatment [13]. High-resolution magic angle spinning (HR-MAS) MRS has proven to be a useful technique for assessment of choline (Cho) metabolism, as it allows detection of individual Cho metabolites in intact tissue specimens. High levels of Cho and phosphocholine (PCho), which are the main contributors to the tCho signal, have been demonstrated in cultured breast cancer cells [14,15], while high levels of glycerophosphocholine (GPC) have been detected in human breast cancer biopsies and xenografts [16-18]. Cho metabolism has been shown to be altered following chemotherapy [19,20], and several enzymes involved in Cho metabolism have been identified as potential drug targets [21,22]. Despite the potential diagnostic value of Cho-containing compounds, the underlying mechanisms causing the alterations in Cho metabolism are not fully understood [23]. Integration of metabolic abnormalities and altered gene expression profiles provides new insights into the underlying regulatory network. Elucidation of the biochemical mechanisms governing Cho metabolism may be useful in the development of prognostic and predictive tools in breast cancer management.

The purpose of this study was to map the metabolomic and transcriptomic characteristics of 34 patient-derived breast cancer xenografts, with a special focus on Cho metabolism. In order to evaluate the clinical relevance of the xenograft models for metabolism studies, human breast cancer biopsies from the corresponding molecular subtypes were analyzed using identical methods.

## Methods

### Xenograft models

Patient-derived breast cancer xenograft models (N = 34) were established at Institute Curie, France (N = 32) or Institute for Cancer Research, Oslo University Hospital (N = 2), either from primary tumor tissue (N = 28), axillary lymph node metastases (N = 4) or metastasis from distant organs (N = 2), as previously described [6-8]. Briefly, primary mammary tumor specimens were implanted into immunodeficient mice receiving estrogen-enriched drinking water. After initial establishment, the tumor tissue from the xenografts was serially transplanted in mice with passage times of two to eight months. Histopathology and immunohistochemistry data from the xenografts was obtained as previously described [6,24]. Twenty nine xenograft tumors were classified as invasive ductal carcinomas (IDC), two were classified as invasive lobular carcinomas (ILC), one as ductal *in situ* carcinoma (DCIS), one as invasive cribriform carcinoma (ICC) and one as micropapillary carcinoma (IMPC). Hormone receptor status of estrogen (ER) and progesterone (PgR) was determined where samples with ≥10% staining cancer cells were considered receptor positive [24]. For the HER2, only membranous staining was interpreted as previously described [25], and protein positivity was defined if ≥65% of the cells were positive. The use of all tissue was evaluated and approved by appropriate ethics research authorities (Norway: Regional Committee for Medical and Health Research Ethics (REC), South East, reference number S-07398a. France: French Ethics Committee, Agreement B75-05-18). Informed written consent was obtained from all but for the two Norwegian patients, who passed away before this issue was addressed. This was evaluated and the need for such consent was waived by the Regional Committee for Medical and Health Research Ethics, South-East Norway. All experiments were conducted according to the regulations of the Federation of European Laboratory Animal Science Association (FELASA).

Tumor tissue was harvested from each of the xenograft models, immediately frozen in liquid nitrogen and stored under cryogenic conditions until analysis. Tumor samples from each model were later divided into two pieces, one for HR-MAS MRS and one for gene expression analysis.

### Human tissue samples

**Figure 1:**
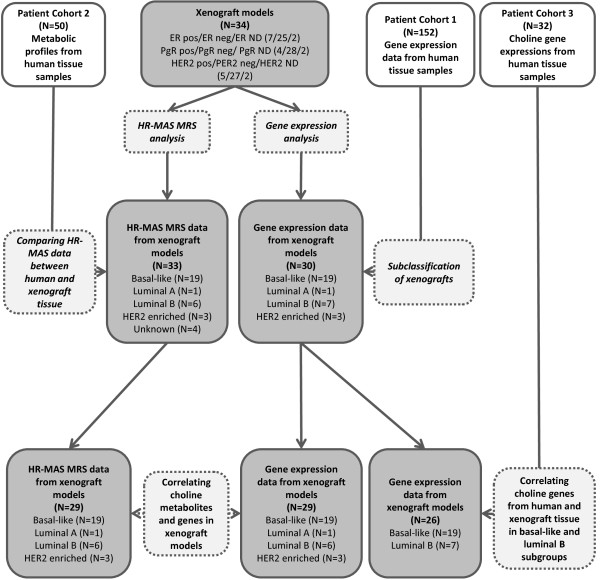
**Flowchart of xenograft samples, human tissue samples and experiments.** The study includes 34 xenograft models (grey rectangles) and tissue samples from three patient cohorts (white rectangles). The experiments performed on the xenografts and human tissue samples are shown by dashed rectangles. ER, estrogen receptor; HER2; human epidermal growth factor receptor 2; ND, not determined; PgR, progesterone receptor.

Three different patient cohorts were included in this study. Human breast cancer biopsies were analyzed using the same methods as for the xenograft models. Gene expression data from breast cancer tissue samples from patients (N = 152) in Cohort 1 (from three different hospitals in the Oslo region, Norway) were used solely to mean center global gene expression data from the xenografts before subclassification. Patient Cohort 2 (N = 50, from two different hospitals in the Trondheim region, Norway) was used to evaluate metabolic characteristics in breast cancer subgroups. The MR spectra from the breast cancer tissue samples were selected from our local spectral database based on ER receptor status. The samples were previously classified as ER positive (N = 37) and ER negative (N = 13) measured by immunohistochemistry (IHC). The ER staining cut-off point was 10% (that is, <10% considered negative). Breast cancer tissue samples from patients (N = 115) in Cohort 3 (patients enrolled at The Norwegian Radium Hospital) were used for comparative analysis of gene expression between human and xenograft samples. This patient material was collected and gene expression analysis performed as previously described in [26,27]. Only basal-like (N = 18) and luminal B samples (N = 14) were selected from the data set, and only expression of genes directly involved in Cho metabolism was used in the analyses. An overview of the xenograft samples, patient cohorts and analyses is shown in Figure 1. The use of all patient materials was approved by Regional Committees for Medical and Health Research Ethics (South East and Central Norway), and informed written consent was obtained from all included patients.

### Breast cancer subclassification using gene expression in xenograft models

#### RNA extraction and microarray hybridization of xenograft tissue

Total RNA from 34 snap frozen xenograft tissue samples was isolated using TRIzol reagent (Invitrogen, Carlsbad, CA, USA) according to the manufacturer’s protocol. Total RNA concentration was measured using NanoDrop (NanoDrop Technologies, Wilmington, DE, USA) and the quality was evaluated using 2100 Bioanalyzer (Agilent Technologies, Waldbronn, Germany). A total of 100 to 125 ng RNA was amplified and labeled with cy3-CTP following the Agilent Low Input Quick Amplification Labeling Kit protocol for One-Color Microarray-Based Gene Expression Analysis. Hybridization was performed according to the manufacturer’s protocol (Agilent One-Color Microarray-Based Gene Expression Analysis v6.5) using 600 ng cy3-labeled cRNA per sample and SurePrint G3 Human Gene Expression 8x60K Microarrays. The microarrays were scanned using Agilent Technologies Microarray Scanner (G2505C). Data were extracted from the scanned images using Feature Extraction software (Agilent Technologies), version 10.7 and protocol GE1-107-Sep09 for mRNA. Four xenograft samples (HBCx-11, HBCx-23, HBCx-29, and HBCx-33) were excluded from further analyses due to poor data quality.

#### Processing, normalization, and molecular subtyping

Raw signals were detrended for multiplicative effects using Agilent’s GenomeAnalyzer and log2 transformed. Data from control probes were excluded, as well as spots that were defined as feature outliers from Feature Extraction due to quality assessment and non-uniform signal distribution. Data were then quantile normalized (R, limma package) and missing values imputed using LLSimpute (R, pcaMethods package). The dataset was generated by additionally averaging the signal intensity of the multiple unique probes for each gene based on GeneSymbol as provided by Agilent in the annotation file. This set included data for 21,851 unique genes on 30 microarrays from 30 breast cancer xenografts.

In order to determine the molecular intrinsic subtype of the xenograft tumors, the gene expression data from the 30 samples were mean centered against a larger, more heterogeneous human breast cancer gene expression dataset of 152 tumors (Cohort 1): for each probe, the mean expression in the reference dataset was subtracted from the probe signal in each of the xenograft samples. Probes from the xenograft dataset were matched to the CloneIDs (CLID) representing the intrinsic genes by corresponding GeneSymbol, obtaining GeneSymbols corresponding to each CLID from the Source tool [28]. For each CLID, if there was more than one matching probe, their expression values were averaged.

The molecular subclassification of the xenografts was determined based on distinct variation in gene expression pattern of 500 'intrinsic’ genes, characteristic for five major molecular breast cancer subtypes. Five expression centroids, calculated for the intrinsic core members of each of the five subclasses [29] were used to determine correlation coefficients between the centroids and each of the 30 xenografts, estimating the xenograft molecular subtype by assigning the subtype which had the highest correlation coefficient to each of the xenografts. The microarray data are available at the Gene Expression Omnibus (GEO) with accession number GSE44666.

### HR-MAS MRS analysis

#### HR-MAS MRS of xenograft tissue

HR-MAS MR spectra from 33 of the xenograft tumor models were acquired. No tumor tissue was available for HR-MAS MRS analysis from one of the models (HBCx-26). Before running the HR-MAS MRS experiments, 3 μL of phosphate-buffered saline (PBS in D_2_O) containing the internal standards trimethylsilyl tetradeuteropropionic acid (TSP, 79.86 mM) and formate (78.80 mM) was added to a disposable insert. The xenograft samples (9.9 ± 2.6 mg) were cut to fit into the disposable insert and placed into a zirconium HR-MAS rotor (4 mm diameter, 80 μL). The HR-MAS MR spectra were acquired using a Bruker Avance III 600 MHz/54 mm US with a ^1^H/^13^C MAS probe with a gradient aligned with the magic angle axis (Bruker Biospin, Rheinstetten, Germany). Samples were spun at 5 kHz, and all experiments were performed at 5°C.

^1^H MR spectra were obtained using a water presaturation sequence (zgpr; Bruker) and a 90° pulse. Water suppression was achieved by irradiation during recycling delay (five seconds). Thirty-two Free Induction Decays (FIDs) were acquired into 32 K points during 3.4 seconds. Total acquisition time was about 4.6 minutes. The FIDs were multiplied with a 0.3Hz exponential line broadening and Fourier transformed with no zero filling. All spectra were phased and baseline corrected. Chemical shifts were calibrated to creatine at 3.04 ppm.

#### HR-MAS MRS of human tissue

Similarly to the xenograft samples, the MR spectra from patient Cohort 2 were acquired at 5°C with a spin rate of 5 kHz. In brief, a 50 μL rotor was filled with buffer containing D_2_O, PBS, TSP (1.37 mM), and formate (10.98 mM). The tissue samples (17.0 ± 4.7 mg) were cut to fit the rotor. ^1^H MR spectra were obtained using a water presaturation sequence (zgpr; Bruker) and a 90° pulse with a Bruker Avance DRX600 spectrometer equipped with a ^1^H/^13^C MAS probe and a gradient aligned with the magic angle axis (Bruker Biospin). Water suppression was achieved by irradiation during recycling delay (three seconds). Thirty-two FIDs were acquired during 2.7 seconds into 32 K and Fourier transformed with no zero filling. Total acquisition time was about 3.8 minutes. The spectra were phased and baseline corrected and chemical shifts were calibrated to creatine at 3.04 ppm.

#### Quantification of metabolites

Pulse length based concentration determination (PULCON) was used for quantification of Cho, PCho and GPC in the xenograft tissue samples. PULCON can be used to measure metabolite concentrations without the use of an internal reference [30]. For quantification of the metabolites in the human tissue samples in Cohort 2, metabolite peak areas of Cho, PCho and GPC were related to the internal standard TSP and sample wet weight. The areas under the Cho, PCho and GPC peaks were determined by curve fitting (PeakFit v 4, Systat Software Inc) using a combination of Gaussian and Lorentzian line-shapes (Voigt function). Two-sample t-tests were performed to assess differences in the metabolite concentrations and ratios between the breast cancer subgroups. The level of significance was set at *P* <0.05.

### Multivariate statistical analysis of MR spectra

For multivariate analysis, the MR spectra from the xenograft samples were converted to ASCII-files, baseline corrected [31] and peak aligned [32]. The spectral area from 3.0 to 4.7 ppm was selected for multivariate analysis. Regions in the spectra with high signals from fatty acids, water and ethanol contaminations were excluded. The MR spectra were normalized and mean centered prior to analysis. Principal component analysis (PCA) was performed (PLS toolbox, Matlab) to visualize the spectral characteristics and to compare the metabolic differences between samples.

### Statistical analysis of gene expression profiles

A total of 55 genes coding for proteins assumed to be directly associated with Cho metabolism were selected. The selection criteria were: 1) a selection of genes involved in the KEGG *Homo sapiens* glycerophospholipid pathway hsa:00564 which have a possible catalytic specificity in this pathway [33]; and 2) genes coding for proteins reported to be involved in Cho transport [34], or 3) involved in degradation of GPC [35]. One gene (*PLA2G4B*) was excluded because it was not found in the gene array. PCA (PLS toolbox, Matlab) was performed on the gene expression data from the 54 selected Cho genes to compare gene expression characteristics between the xenograft samples. The gene data were mean normalized and mean centered prior to analysis.

### Correlation analysis between choline metabolites and genes in xenografts

In order to identify genes directly associated with regulation of Cho metabolism, the correlation between the 54 selected Cho genes and the concentrations of three metabolites, Cho, PCho and GPC was calculated using Pearson’s correlation test. This analysis was repeated separately for the basal-like and luminal B subgroups, in order to evaluate differences in regulation of Cho metabolism between these subtypes.

### Correlation analysis between choline genes in xenografts and human tissue

In order to assess if the panel of xenograft models was representative for human disease, the average difference in expression of the 54 Cho-related genes between basal-like and luminal B subtype samples was computed. One gene (*ASPG*) was excluded because it was not found in the gene array from the patient tissue samples. Fifty three of the genes were present in the dataset both for the patient samples (Cohort 3) and the xenografts. In order to validate the relevance of the xenograft models, a Pearson correlation test was performed between genes from the patient and the xenograft subtypes. For all these analyses, the level of statistical significance was defined at fdr <0.1.

## Results

### The majority of xenograft tumors were classified as basal-like and luminal B subtypes

The expression of ER, PgR and HER2 receptors of the 34 xenograft models was previously determined by IHC and real-time quantitative reverse transcription-PCR (RT-PCR) methods [6,7,24]. In order to further characterize these models, the gene expression analysis was carried out with the Agilent microarray platform. Nineteen samples were classified as basal-like, one as luminal A, seven as luminal B, and three samples as HER2 enriched. Five out of seven luminal B tumors had positive ER status, while all basal-like tumors were classified as ER negative. Two of the three HER2 enriched tumors were characterized by positive HER2 membrane staining. A detailed description of each xenograft models is given in Table 1.

**Table 1:** Molecular characteristics of the 34 xenografts

	**Intrinsic molecular subtype**					**Receptor status**			**Histological classification**					**Metastasis**	
**Model**	**Basal-like**	**Luminal B**	**Luminal A**	**HER2 enriched**	**Unknown**	**ER pos.**	**PgR pos.**	**HER2 pos.**	**IDC**	**ILC**	**ICC**	**DCIS**	**IMPC**	**Node**	**Distant**
HBCx-1	X								X						
HBCx-4B	X								X					X	
HBCx-7	X									X					X
HBCx-8	X								X						
HBCx-9	X								X						
HBCx-10	X								X						
HBCx-12A	X								X						
HBCx-12B	X								X					X	
HBCx-14	X								X						
HBCx-15	X								X						
HBCx-16	X								X						
HBCx-17	X								X						
HBCx-24	X								X						
HBCx-27	X								X						
HBCx-28	X								X						
HBCx-30	X								X						
HBCx-39	X								X						
HBCx-41	X							X					X		
MAS98.12	X								X						
HBCx-3		X				X			X						
HBCx-5		X				X		X	X					X	
HBCx-19		X								X					X
HBCx-26		X				X		X	X						
HBCx-31		X							X						
HBCx-34		X				X	X		X						
MAS98.06		X				X	X		X						
HBCx-21			X			X	X					X			
HBCx-13A				X				X	X						
HBCx-13B				X				X	X					X	
HBCx-40				X					X						
HBCx-11					X						X				
HBCx-23					X				X						
HBCx-29					X	X	X	X	X						
HBCx-33					X				X						
Total numbers	19	7	1	3	4	7	4	6	29	2	1	1	1	4	2

The table reports on intrinsic molecular subtype, receptor status, histological classification and metastatic potential.

DCIS, ductal *in citu* carcinoma; ER, estrogen receptor; HER2, human epidermal growth factor receptor 2; ICC, invasive cribriform carcinoma; IDC, invasive ductal carcinoma; ILC, invasive lobular carcinoma; IMPC, invasive micropapillary carcinoma; PgR, progesterone receptor

### Metabolic characterization

#### PCho/GPC ratio in subtypes of breast cancer

**Figure 2:**
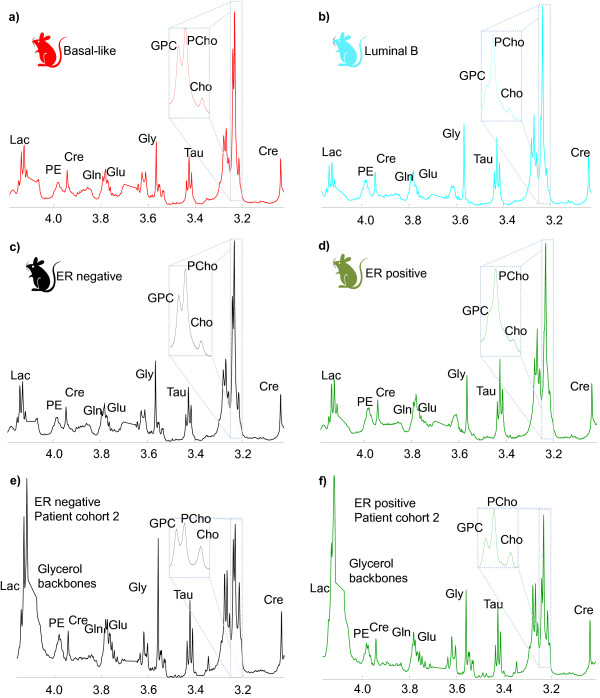
**Mean MR spectra of different breast cancer subtypes.** MR spectra of **a)** basal-like (N = 19), **b)** luminal-B (N = 6), **c)** ER negative (N = 27) and **d)** ER positive (N = 6) xenograft samples, and of **e)** ER negative (N = 13), and **f)** ER positive (N = 37) breast cancer tissue samples from patients. Abbreviations: Lac: lactate, PE: phosphoethanolamine, Cre: creatine, Gln: glutamine, Glu: glutamate, Gly: glycine, Tau: taurine, GPC: glycerophosphocholine, PCho: phosphocholine, Cho: choline.

Mean MR spectra from the basal-like (N = 19) and luminal B (N = 6) xenograft samples are shown in Figure 2a and 2b, respectively. The luminal-like samples were characterized by a high PCho/GPC ratio (2.5 ± 0.9), and only one sample with GPC > PCho. The basal-like samples were characterized with a higher variation in the PCho/GPC ratio (1.6 ± 1.2) and the majority of the samples with GPC > PCho were present in this group (8 of 19 MR spectra). Figure 2c and 2d show mean MR spectra of xenograft samples from ER negative (N = 27) and ER positive (N = 6) xenograft samples. Mean MR spectra from the ER negative (N = 13), and ER positive (N = 37) samples from patient Cohort 2 are shown in Figure 2e and 2f, respectively. A higher PCho/GPC level was observed in the ER positive (2.0 ± 1.1) compared to the ER negative (1.0 ± 0.7) human tissue samples (*P* < 0.01). PCho/GPC box plots for the different subgroups are shown in Additional file 1. In one MR spectrum from the xenografts, and two MR spectra from patients, the measured PCho/GPC ratios were excluded since they were classified as outliers by the extreme studentized deviate analysis (*P* < 0.05) [36]. All outliers had a high PCho/GPC ratio.

#### Multivariate analysis reveals metabolic differences between subtypes of breast cancer

**Figure 3:**
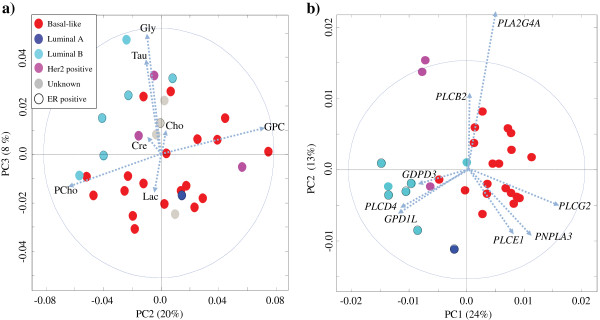
**Score plot and loading profiles (dotted arrows) from PCA of HR-MAS MR spectra and expression of genes encoding proteins in the choline pathway in the xenograft tissue samples. a)** Bi-plot of PC2 and PC3 from HR-MAS MR spectra (N = 33). **b)** Bi-plot of PC1 and PC2 for selected genes contributing to choline metabolism. HR-MAS MR, high resolution magic angle spinning magnetic resonance; PCA, principal component analysis.

PCA was performed on the MR spectra from the xenografts to investigate the metabolic characteristics of the models. Figure 3a shows the bi-plot (combined score and loading plot). Most of the luminal B samples are clustered in the top left corner of the score plot with a low principal component 2 (PC2) score and a high PC3 score. Samples with low PC2 scores are characterized by higher levels of PCho, creatine, taurine, glycine and lactate, and lower levels of GPC and Cho, compared to samples with a high PC2 score. PC2 accounted for 20% of the variance between the samples, and the loading profile indicates a large contribution from the amount of PCho and GPC. PC3 explained 8% of the variance between the samples. Samples with high PC3 scores are characterized with higher levels of glycine, taurine, GPC, Cho and creatine, and lower levels of lactate and PCho. PC1 showed that the largest variance between the samples (43%) was caused by variations in lipid content (data not shown). No clustering of breast cancer subgroups was found in PC1.

### Gene expression analysis of the xenograft models and human tissue samples

#### Multivariate analysis reveals differences in gene expression of genes involved in choline metabolism between subtypes of breast cancer

In order to map the gene expression characteristics of the Cho metabolism pathway in the xenograft models, a PCA was performed. Figure 3b shows the bi-plot from PCA based on the expression data of the 54 genes in the Cho pathway. Most of the luminal B samples are clustered in the bottom left corner of the score plot with a low PC1 and a low PC2 score. These samples are characterized with a high expression of glycerol-3-phosphate dehydrogenase 1-like (*GPD1L)*, phospholipase C, delta 4 (*PLCD4)* and glycerophosphodiester phosphodiesterase domain containing 3 (*GDPD3)*. Most of the basal-like samples are clustered to the right of the score plot with a high PC1 score. These samples are characterized with high expression of phospholipase C, gamma 2 (*PLCG2)*, phospholipase domain containing 3 (*PNPLA3)*, phospholipase C, epsilon 1 (*PLCE1)* and phospholipase A2, group IVA (*PLA2G4A)*.

#### Correlations between choline metabolites and gene expressions in xenograft models

**Figure 4:**
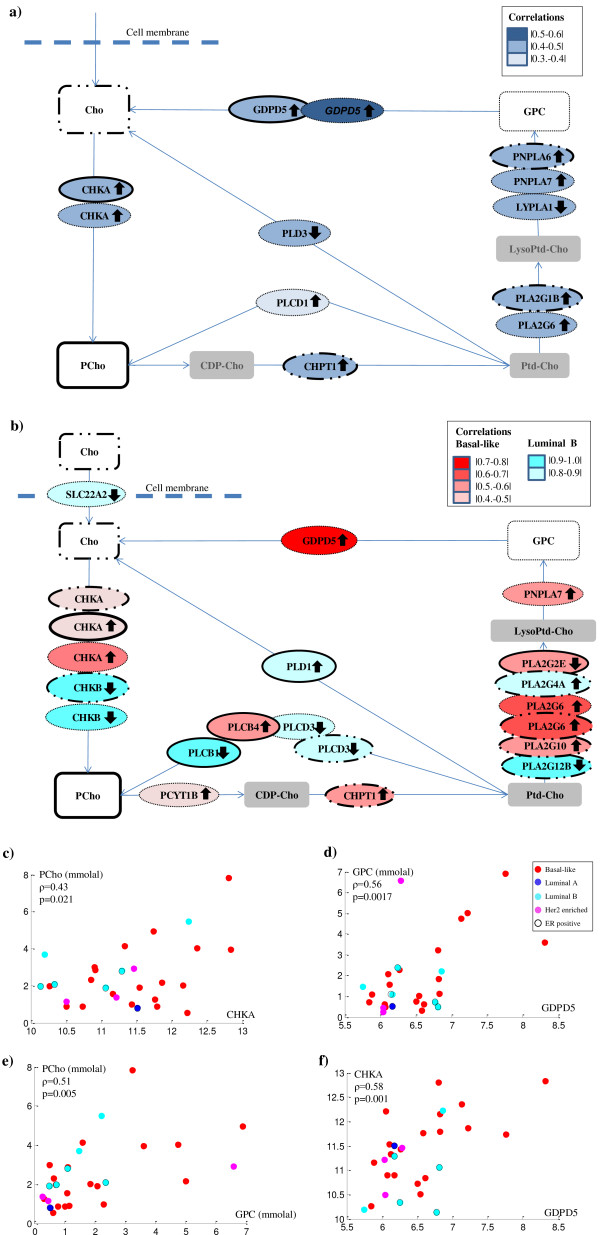
**Major metabolites and genes contributing to the choline metabolism pathway. a)** Genes (oval) in choline metabolism being either positively (arrows upwards) or negatively (arrows downward) correlated with Cho, PCho or GPC (rectangles) in the xenograft models. High expressions of *CHPT1, PLA2G1B* and *PNPLA6* (marked with dots and solid outlines) are associated with high levels of Cho*.* High expressions of *CHKA* and *GDPD5* (black outlines) are associated with high levels of PCho. High expressions of *CHKA*, *GDPD5*, *PLA2G6*, *PLCD1* and *PNPLA7*, and low expressions of *LYPLA1*, and *PLD3* (dashed outlines) are associated with high levels of GPC. **b)** Genes (oval) that are either positively or negatively correlated with Cho, PCho or GPC (rectangles) in the basal-like, and luminal B subgroup. In the basal-like subgroup (red), high expressions of *CHKA, CHPT1, PLA2G10* and *PLA2G6* (dots and solid outlines) are associated with high levels of Cho. High expressions of *CHKA* and *PCLB4,* and a low expression of *PLA2G2E* (solid outlines) are associated with high levels of PCho. High expressions of *CHKA*, *GDPD5*, *PCYT1B*, *PLA2G6* and *PNPLA7* (dashed outlines) are associated with high levels of GPC. In the luminal B subgroup (cyan) a high expression of *PLA2G4A*, and low expressions of *CHKB*, *PLA2G12A* and *PLCD3* (dots and solid outlines) are associated with high levels of Cho. A high expression of *PLD1*, and a low expression of *PLCB1* (solid outlines), are associated with high levels of PCho. In addition, low levels of *CHKB*, *PLCD3* and *SLC22A2* (dashed outlines) are associated with high GPC levels. **c-f)** Scatter plots of correlation between **c)** PCho and *CHKA*, **d)** GPC and *GDPD5*, **e)** PCho and GPC and **f)** *GDPD5* and *CHKA*. ρ: Pearson’s correlation coefficient, Gene expression: normalized log 2 transformed.

To detect associations between the expression of Cho genes and the concentrations of Cho, PCho and GPC, a correlation analysis was performed. Across all xenograft models, ten of the 54 genes contributing to Cho metabolism were found to be correlated with Cho, PCho or GPC concentration (*P* <0.05), as shown in Table 2 and Figure 4a. Cho was positively correlated with the expression of *CHPT1,* phospholipase A2, group IB *(PLA2G1B)* and patatin-like phospholipase domain containing 6 *(PNPLA6).* PCho was positively correlated with expression of choline kinase α (*CHKA*) and glycerophosphodiester phosphodiesterase domain containing 5 (*GDPD5*). GPC was positively correlated with *CHKA*, *GDPD5*, phospholipase A2 group VI (*PLA2G6*), phospholipase C, delta 1 (*PLCD1*) and patatin-like phospholipase domain containing 7 (*PNPLA7*), and negatively correlated with lysophospholipase 1 (*LYPLA1*) and phospholipase D family, member 3 (*PLD3*). In addition, a high expression of *CHKA* was also found to be correlated with high expression of *GDPD5*, and PCho concentration was positively correlated to GPC concentration. Scatter plots of correlations between metabolites and gene expressions are shown in Figure 4c- 4f.

**Table 2:** **Correlation coefficients (ρ) and levels of significance (*P*) between concentration of Cho, PCho, and GPC, and expression of genes contributing in the choline metabolism pathway**

**Subgroup**	**Metabolite**	**Gene**	**ρ**	***P*-value**
All samples (N = 30)	Cho	*CHPT1*	0.49	0.008
		*PLA2G1B*	0.43	0.021
		*PNPLA6*	0.41	0.028
	PCho	*CHKA*	0.43	0.021
		*GDPD5*	0.38	0.041
	GPC	*CHKA*	0.44	0.017
		*GDPD5*	0.56	0.002
		*LYPLA1*	-0.40	0.030
		*PLA2G6*	0.43	0.021
		*PLCD1*	0.37	0.045
		*PLD3*	-0.42	0.024
		*PNPLA7*	0.39	0.035
Basal-like (N = 19)	Cho	*CHKA*	0.46	0.049
		*CHPT1*	0.50	0.029
		*PLA2G10*	0.53	0.021
		*PLA2G6*	0.61	0.006
	PCho	*CHKA*	0.47	0.044
		*PLA2G2E*	-0.52	0.024
		*PLCB4*	0.51	0.027
	GPC	*CHKA*	0.52	0.023
		*GDPD5*	0.73	<0.001
		*PCYT1B*	0.49	0.035
		*PLA2G6*	0.67	0.002
		*PNPLA7*	0.52	0.021
Luminal B (N = 6)	Cho	*CHKB*	-0.96	0.002
		*PLA2G12B*	-0.91	0.012
		*PLA2G4A*	0.86	0.029
		*PLCD3*	-0.86	0.027
	PCho	*PLCB1*	-0.91	0.011
		*PLD1*	0.89	0.017
	GPC	*CHKB*	-0.97	0.001
		*PLCD3*	-0.81	0.049
		*SLC22A2*	-0.86	0.030

#### Correlation between choline metabolites and gene expression within the basal-like and luminal B subgroups

The correlation analysis was then performed separately for basal-like and luminal B xenograft samples, in order to identify potential differences in regulation of Cho metabolism between these subtypes. When considering only the basal-like xenograft samples, nine genes were found to be correlated to either Cho, PCho or GPC, as shown in Figure 4b) and Table 2. Cho was positively correlated with *CHKA, CHPT1,* phospholipase A2, group X (*PLA2G10*) and *PLA2G6*. PCho was positively correlated with phospholipase C, beta 4 *(PLCB4)* and *CHKA*, and negatively correlated with *PLA2G2E*. GPC was positively correlated with *CHKA*, *GDPD5*, phosphate cytidylyltransferase 1, Cho, beta (*PCYT1B)*, *PLA2G6* and *PNPLA7*. In the luminal B subgroup, Cho, PCho and GPC were correlated with seven Cho genes. Cho was positively correlated with *PLA2G4A*, and negatively correlated with choline kinase beta (*CHKB*), phospholipase A2, group XIIB (*PLA2G12B*) and phospholipase C, delta 3 (*PLCD3*). PCho was found to be positively correlated with phospholipase C, delta 1 (*PLD1)*, and negatively correlated with phospholipase C, beta 1 *(PLCB1)*, while GPC was found to be negatively correlated with solute carrier family 22, member 2 *(SLC22A2)*, *CHKB* and *PLCD3*. A list of all correlations between Cho, PCho, and GPC versus Cho genes is shown in Additional file 2.

#### Correlations between choline genes in xenografts and human tissue

**Figure 5:**
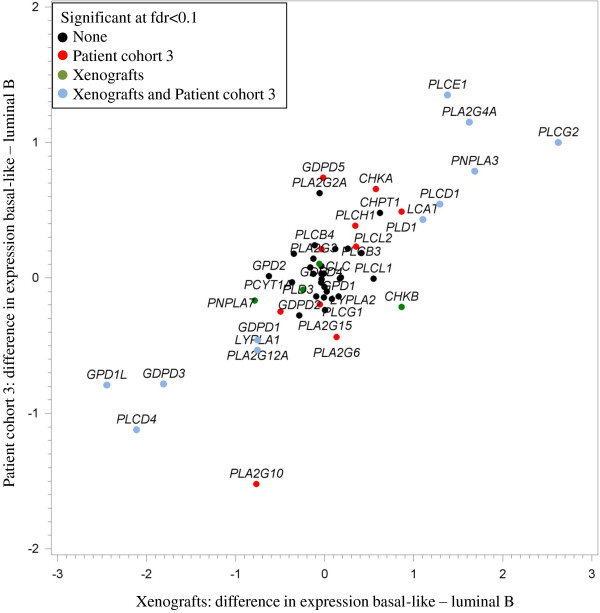
**Comparison of choline gene expressions between basal-like and luminal B xenografts and human tissue samples (Cohort 3).** Blue dots show genes being differentially expressed for basal-like versus luminal B, both for the xenografts and human tissue samples. Green dots show genes being differentially expressed for basal-like versus luminal B for the xenograft models only, while red dots show genes differentially expressed for basal-like versus luminal B subgroups for the human tissue samples only. Black dots are genes not being differentially expressed between basal-like and luminal B samples in neither the xenografts nor the human tissue samples.

The differential expression of Cho genes between the basal-like and luminal B subtypes was studied both in xenograft and human tissue samples. A strong correlation (ρ = 0.79, *P* <1.3e-12) was observed when expression of genes in the Cho metabolism pathway was compared in tissue samples from xenografts and breast cancer patients (Cohort 3). Figure 5 shows differences in gene expression between basal-like and luminal B samples for xenografts and human tissue samples. The plot demonstrates that six genes: *PLCG2*, *PLCE1*, *PLA2G4A*, *PNPLA3*, *PLCD1* and lecithin-cholesterol acyltransferase (*LCAT)*, were significantly higher expressed in basal-like compared to luminal B samples, both for the xenografts and human tissue samples. Five genes: *PLCD4*, *GPD1L*, *GDPD3*, phospholipase A2, group XIIA (*PLA2G12A)* and *LYPLA1,* were significantly lower expressed in basal-like compared to luminal B samples, for both the xenografts and human tissue samples. A list of all gene expressions, differences in gene expressions between basal-like and luminal B tissue samples, *P*-values and fdr-values for the patients and the xenograft samples is given in Additional file 3.

## Discussion

In this study, metabolic and gene expression profiles of 34 patient-derived breast cancer xenograft models have been characterized and compared with patient breast cancer samples. The majority of the xenograft models was classified as basal-like and had a triple-negative receptor status. The gene expression profiling was consistent with IHC assessments previously reported for these tumors [7,24]. The luminal B/ER positive xenograft samples were characterized by a high PCho/GPC ratio. For the basal-like subgroup, a larger variation in the PCho/GPC ratio was found within the xenograft samples. The metabolic profiles of the xenografts corresponded well with the profiles obtained from human breast cancer tissue. The expression of genes associated with Cho metabolism was found to be different in luminal B and basal-like xenograft models, which also were in accordance with findings in the corresponding subgroups of human breast tumor tissue samples.

Eighteen of twenty four triple-negative samples were classified as basal-like cancers. These results corresponded well with findings from other studies, since approximately 90% of triple-negative breast carcinomas are classified as basal-like [29,37]. In addition, expression of estrogen receptors is a known feature of luminal A and B subtypes of breast cancer [38], and all of the ER positive xenografts were found to be luminal A or B. Overall, the association between histopathological characteristics and intrinsic molecular subclassification was in accordance with previously published data [39]. This confirms that the molecular subclassification of xenografts reflects the typical characteristics seen in human disease despite the presence of mouse stromal cells and thereby potentially different tumor/host interaction than in human tumors.

In concordance with the high PCho/GPC ratio in the luminal-like/ER positive xenografts, significantly higher PCho/GPC levels were found in the ER positive versus ER negative samples from breast cancer patients. These results are in agreement with findings in other studies of human breast cancer tissue and xenografts [17,40] but do not correspond to results from *in vitro* studies. Studies of a panel of cultured cell lines have suggested that malignancy is associated with high PCho and low GPC levels [14]. High GPC levels found *in vivo*, both in xenograft tissue and clinical samples, suggests that this hypothesis has to be refined. Due to the discrepancy between *in vitro* and *in vivo* data, it is tempting to speculate that microenvironmental factors may play a role in the *in vivo* regulation of Cho metabolism [41,42]. In addition, there is a possibility that high GPC concentrations could be linked to differences in driver mutations between ER positive and ER negative tumors. Luminal-like breast cancer is strongly associated with ER expression, and metabolism is likely tightly regulated by ER-mediated mechanisms. In basal-like breast cancer, the impact of ER (and HER2) –mediated signaling plays a smaller role. The activity in other signaling pathways, such as PI3K and MAPK, is, therefore, comparatively more important, resulting in a more heterogeneous metabolic profile.

The results from the gene expression profiles indicated that the genes involved in Cho metabolism were differentially expressed in luminal B compared to basal-like xenograft samples. The luminal B xenograft samples were found to have a higher expression of *PLCD4*, *GDPD3* and *GPD1L*, while the basal-like samples were characterized with higher expression of *PLCG2*, *PNPLA3* and *PLCE1*. In addition, the concentrations of Cho, PCho and GPC were correlated with the expression of different genes in different breast cancer subgroups. This suggests that luminal B and basal-like breast cancer may have different mechanisms regulating Cho metabolism. The concordance between the gene expression profiles from the xenograft models and breast cancer tissue samples confirmed the assumption that these xenografts are representative models of human breast cancer.

Although tCho is proposed as an *in vivo* biomarker in breast cancer, the regulation of Cho metabolism is not fully understood. The current consensus is that the transport of Cho into cancer cells is increased compared to normal cells. *In vitro*, increased expression of various Cho transporter proteins has been demonstrated, and *in vivo* PET studies using [^11^C]-choline or [^18^ F]-fluorocholine have indicated increased uptake of Cho both in preclinical models and clinical studies [43-46]. Furthermore, it has been shown that *CHKA* is upregulated in several cancers, and that *CHKA* expression correlates with PCho concentration *in vitro*[45,47]. In our study, the positive correlation between *CHKA* expression and PCho concentration in xenograft tumors was confirmed. The regulation of GPC is poorly understood [48], which is a challenge as this metabolite contributes significantly to the tCho signal measured by *in vivo* MRS. Several studies have demonstrated that GPC may be a potential biomarker for response to treatment [21,49-52], and it is, therefore, necessary to elucidate the mechanisms responsible for regulating GPC concentration *in vivo*. We found a positive correlation between *CHKA* and GPC concentration, which is not surprising as PCho and GPC concentrations are positively correlated. This suggests that malignant transformation and upregulation of *CHKA* leads to a general increase in PtdCho turnover, which is reflected by a high concentration of both precursor (PCho) and degradation products (GPC) of this cell membrane component. A positive correlation between *GDPD5* and PCho concentration was also found which is in accordance with previous studies suggesting that *GDPD5* may be upregulated in ER negative breast cancer [53]. As GDPD5 has been suggested to catalyze GPC degradation, the positive correlation between *GDPD5* and GPC concentration was not anticipated. These results suggest that *GDPD5* may be a general marker for abnormal Cho metabolism, but not necessarily regulating GPC concentration. This interpretation is further supported by the positive correlation between expression of *CHKA* and *GDPD5*.

Various phospholipase enzymes are involved in degradation of PtdCho to GPC, PCho, and Cho but the roles of the various isoforms are still not fully elucidated. Several phospholipases are upregulated in cancer compared to normal tissue [54-57], but their impact on the concentration of Cho-containing metabolites is poorly understood [58]. The results of this study indicate that expression of phospholipases varies significantly between the xenograft models within the different breast cancer subgroups. However, the large number of phospholipase isoforms and their complex biology makes it difficult to interpret the significance of these differences. Numerous studies have demonstrated complex and often reciprocal interactions between oncogenic signaling pathways and enzymes involved in Cho metabolism [23]. Several enzymes involved in Cho metabolism, including CHK, PLC, PLD, and PLA2, have been shown to be affected by RAS-mediated signaling [59,60]. MYC and HIF1 have also been shown to be involved in the regulation of CHKA [61]. When we observe differences in the Cho metabolic and gene expression profiles between cancer subtypes, it may be caused by specific oncogenic signaling pathways that are more frequently upregulated in some subtypes.

More than 10 years after the first report on molecular fingerprints in breast cancer [2], there are still active discussions on the optimal strategy for subtyping breast cancer. Several research groups advocate an integrated approach, where data from several –omics platforms are combined for identification of clinically relevant subtypes [62,63]. Since MR metabolomics reflect tumor microenvironment to a larger degree than other –omics techniques, it can contribute to improved understanding of the underlying biology in different breast cancer subtypes. Including metabolic profiles in the criteria for novel breast cancer subtypes may, therefore, bring us closer to personalized breast cancer treatment.

In this study, gene expression profiling and metabolomic analysis of 34 patient-derived xenograft models demonstrated significant difference between luminal B and basal-like breast cancer. Similar patterns both in metabolic profiles and expression of Cho genes were found in the xenograft models and human breast cancer with corresponding molecular subtype. This panel of patient-derived xenograft models, therefore, represents a unique and valuable tool for studies of molecular properties associated with sensitivity or resistance to chemotherapy or targeted anticancer drugs. It also allows further studies of the unique biology of the different subtypes of breast cancer, which may be important for future clinical applications based on molecular fingerprints.

## Conclusion

HR-MAS MRS and gene expression analyses demonstrated that the amount of Cho, PCho and GPC correlated with the expression of several genes, including *CHKA* and *GDPD5*, in the Cho metabolism pathway in tissue samples from patient-derived breast cancer xenografts representing luminal-like, basal-like and HER2 enriched breast cancer. High PCho/GPC ratios were observed for the luminal-like samples, while a larger variation in the PCho/GPC ratios was observed for the basal-like samples. These results corresponded well with the Cho profiles of human breast cancer samples where a significantly higher PCho/GPC level was found in ER positive compared to ER negative cancers. Both the Cho metabolic profiles and the expression of genes involved in the Cho metabolism pathway differed between luminal B and basal-like xenografts. Similar differences were found in human breast cancer samples, and the differential gene expression between basal-like and luminal B subtypes was correlated strongly in xenografts and human samples. The amount of Cho, PCho and GPC was also correlated to the expression of different Cho genes in the luminal B compared to the basal-like subgroup. Differences in the Cho metabolic and gene expression profiles between cancer subtypes can be caused by specific oncogenic signaling pathways that are more frequently upregulated in some subtypes. The findings in this study indicate that the panel of patient-derived xenografts is representative of human breast cancer, and may be valuable for further exploration of subtype-specific metabolic and transcriptomic traits. In addition, the models are relevant for studies of targeted anticancer drugs and molecular properties associated with sensitivity and resistance to chemotherapy.

## Abbreviations

*CHKA*: choline kinase alpha; *CHKB*: choline kinase beta; Cho: choline; *CHPT1*: choline phosphotransferase 1; Cre: creatine; DCIS: ductal *in situ* carcinoma; ER: estrogen receptor; FID: Free Induction Decay; *GDPD3*: glycerophosphodiester phosphodiesterase domain containing 3; *GDPD5*: glycerophosphodiester phosphodiesterase domain containing 5; Gln: glutamine; Glu: glutamate; Gly: glycine; GPC: glycerophosphocholine; *GPD1L*: glycerol-3-phosphate dehydrogenase 1-like; HER2: human epidermal growth factor receptor 2; HR-MAS MRS: high resolution magic angle spinning magnetic resonance spectroscopy; ICC: invasive cribriform carcinoma; IDC: invasive ductal carcinomas; IHC: immunohistochemistry; ILC: invasive lobular carcinomas; IMPC: micropapillary carcinoma; Lac: lactate; *LCAT*: lecithin-cholesterol acyltransferase; *LYPLA1*: lysophospholipase 1; PBS: phosphate-buffered saline; PC1,2,3: principal component 1,2,3; PCA: principal component analysis; PCho: phosphocholine; *PCYT1B*: phosphate cytidylyltransferase 1, choline, beta; PE: phosphoethanolamine; PgR: progesterone receptor; *PLA2G10*: phospholipase A2, group X; *PLA2G12A*: phospholipase A2, group XIIA; *PLA2G12B*: phospholipase A2, group XIIB; *PLA2G1B*: phospholipase A2, group IB; *PLA2G2E*: phospholipase A2, group IIE; *PLA2G4A*: phospholipase A2, group IVA; *PLA2G6*: phospholipase A2, group VI; *PLCB1*: phospholipase C, beta 1; *PLCB4*: phospholipase C, beta 4; *PLCD1*: phospholipase C, delta 1; *PLCD3*: phospholipase C, delta 3; *PLCD4*: phospholipase C, delta 4; *PLCE1*: phospholipase C, epsilon 1; *PLCG2*: phospholipase C, gamma 2; *PLD1*: phospholipase C, delta 1; *PLD3*: phospholipase D family, member 3; *PNPLA3*: patatin-like phospholipase domain-containing protein 3; *PNPLA6*: patatin-like phospholipase domain containing 6; *PNPLA7*: patatin-like phospholipase domain containing 7; *SLC22A2*: solute carrier family 22, member 2; Tau: taurine; TSP: trimethylsilyl tetradeuteropropionic acid

## Competing interests

The authors declared they have no competing interests.

## Authors’ contributions

MTG was involved in the study design, acquired and analyzed the HR-MAS MRS data and drafted the manuscript. NS performed the gene expression analysis, interpreted the microarray data and was involved in drafting of the manuscript. SAM was involved in the study design and in drafting of the manuscript. EAR performed statistical analyses and interpreted the data. EB performed gene expression analysis and interpreted microarray data. AK coordinated the xenograft samples. BS was involved in the HR-MAS MRS protocol and supervised the analyses. TFB was involved in the study design and in statistical analyses. ALBD contributed with expertise in molecular biology techniques. GMM and OE were involved in the coordination of xenografts. TS was involved the study design and in the interpretation of the microarray data. EM was responsible for the establishment of the xenograft models and delivery of samples. ISG designed and coordinated the study. All authors critically revised, read and approved the final manuscript.

## Supplementary Material

Additional file 1 PCho/GPC levels for different subgroups of breast cancer xenografts and human tissue samples. Click here for file

Additional file 2: Table S1 Correlation coefficients and *P*-values between Cho, PCho, and GPC concentrations, and expressions of genes contributing in choline metabolism for all samples (N = 29). All genes and metabolites having a significant correlation (P <0.05) are emphasized in bold. *: false positive, found by visual inspection. **Table S2.** Correlation coefficients and *P*-values between Cho, PCho, and GPC concentrations, and expressions of genes contributing in choline metabolism for the basal-like tissue samples (N = 19). All genes and metabolites having a significant correlation (*P* <0.05) are emphasized in bold. **Table S3.** Correlation coefficients and *P*-values between Cho, PCho, and GPC concentrations, and expressions of genes contributing in choline metabolism for the luminal B tissue samples (N = 6). All genes and metabolites having a significant correlation (*P* <0.05) are emphasized in bold. Click here for file

Additional file 3: Table S4 Mean choline gene expressions for basal-like and luminal B, difference in expression basal-like-luminal B, *P*-values and FDR values, for patient tissue samples and xenografts. All genes being significantly differently expressed for basal-like – luminal B (fdr <0.1) are emphasized in bold. Click here for file
