# Supplementary figures and images for: Interplay of choline metabolites and genes in patient-derived breast cancer xenografts
Source: Breast Cancer Res. 2014 Jan 21;16(1):R5. doi: 10.1186/bcr3597 (PMC3978476; doi:10.1186/bcr3597)

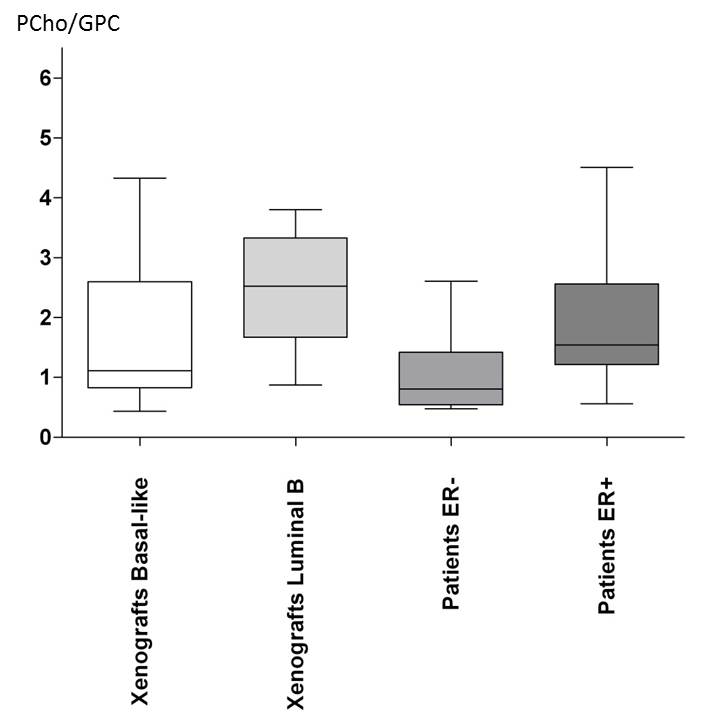

Supplement: Additional file 1 — PCho/GPC levels for different subgroups of breast cancer xenografts and human tissue samples. [file bcr3597-S1.jpeg]
