# Supplementary material for: Interplay of choline metabolites and genes in patient-derived breast cancer xenografts
Source: Breast Cancer Res. 2014 Jan 21;16(1):R5. doi: 10.1186/bcr3597 (PMC3978476; doi:10.1186/bcr3597)
Supplement: Additional file 2: Table S1 — Correlation coefficients and P-values between Cho, PCho, and GPC concentrations, and expressions of genes contributing in choline metabolism for all samples (N = 29). All genes and metabolites having a significant correlation (P <0.05) are emphasized in bold. *: false positive, found by visual inspection. Table S2. Correlation coefficients and P-values between Cho, PCho, and GPC concentrations, and expressions of genes contributing in choline metabolism for the basal-like tissue samples (N = 19). All genes and metabolites having a significant correlation (P <0.05) are emphasized in bold. Table S3. Correlation coefficients and P-values between Cho, PCho, and GPC concentrations, and expressions of genes contributing in choline metabolism for the luminal B tissue samples (N = 6). All genes and metabolites having a significant correlation (P <0.05) are emphasized in bold. [file bcr3597-S2.doc]

# Additional file 2:

Table 1:

| Gene | Cho |  | PCho |  | GPC |  |
| --- | --- | --- | --- | --- | --- | --- |
|  | ρ | p-value | ρ | p-value | ρ | p-value |
| *ASPG* | 0.01 | 0.955 | -0.19 | 0.323 | -0.01 | 0.962 |
| *CHKA* | 0.29 | 0.124 | **0.43** | **0.021** | **0.44** | **0.017** |
| *CHKB* | -0.23 | 0.226 | -0.15 | 0.439 | 0.08 | 0.662 |
| *CHPT1* | **0.49** | **0.008** | 0.12 | 0.534 | 0.15 | 0.444 |
| *CLC* | 0.08 | 0.685 | 0.09 | 0.647 | -0.02 | 0.909 |
| *GDPD1* | 0.08 | 0.668 | 0.05 | 0.808 | 0.00 | 0.994 |
| *GDPD2* | -0.23 | 0.229 | -0.04 | 0.825 | -0.24 | 0.205 |
| *GDPD3* | -0.04 | 0.850 | -0.19 | 0.334 | -0.02 | 0.922 |
| *GDPD4* | 0.27 | 0.150 | 0.23 | 0.236 | 0.28 | 0.147 |
| *GDPD5* | 0.29 | 0.122 | **0.38** | **0.041** | **0.56** | **0.002** |
| *GPD1* | 0.21 | 0.272 | -0.12 | 0.547 | 0.03 | 0.882 |
| *GPD1L* | -0.11 | 0.564 | -0.01 | 0.970 | -0.08 | 0.688 |
| *GPD2* | 0.04 | 0.849 | -0.02 | 0.920 | -0.14 | 0.479 |
| *LCAT* | -0.33 | 0.081 | -0.15 | 0.424 | -0.03 | 0.897 |
| *LYPLA1* | -0.12 | 0.552 | -0.08 | 0.692 | **-0.40** | **0.030** |
| *LYPLA2* | 0.04 | 0.846 | -0.21 | 0.263 | 0.09 | 0.627 |
| *PCYT1A* | 0.28 | 0.143 | 0.04 | 0.831 | 0.14 | 0.463 |
| *PCYT1B* | 0.12 | 0.548 | 0.12 | 0.543 | 0.33 | 0.081 |
| *PLA2G10* | 0.13 | 0.509 | -0.08 | 0.695 | 0.14 | 0.465 |
| *PLA2G12A* | 0.06 | 0.777 | -0.10 | 0.613 | -0.17 | 0.366 |
| *PLA2G12B* | -0.22 | 0.241 | -0.18 | 0.362 | 0.15 | 0.423 |
| *PLA2G15* | -0.28 | 0.137 | -0.27 | 0.155 | -0.07 | 0.701 |
| *PLA2G1B* | **0.43** | **0.021** | 0.10 | 0.607 | 0.04 | 0.856 |
| *PLA2G2A* | -0.05 | 0.780 | 0.02 | 0.913 | **0.451*** | **0.014*** |
| *PLA2G2D* | -0.33 | 0.078 | -0.04 | 0.855 | -0.16 | 0.410 |
| *PLA2G2E* | 0.16 | 0.394 | -0.25 | 0.186 | -0.11 | 0.567 |
| *PLA2G2F* | -0.01 | 0.954 | 0.12 | 0.550 | 0.12 | 0.526 |
| *PLA2G3* | 0.07 | 0.722 | 0.09 | 0.650 | 0.20 | 0.309 |
| *PLA2G4A* | -0.04 | 0.835 | -0.33 | 0.076 | -0.25 | 0.198 |
| *PLA2G5* | -0.13 | 0.516 | 0.00 | 0.987 | 0.24 | 0.214 |
| *PLA2G6* | 0.30 | 0.110 | 0.10 | 0.622 | **0.43** | **0.021** |
| *PLCB1* | -0.27 | 0.164 | -0.28 | 0.139 | -0.31 | 0.100 |
| *PLCB2* | -0.04 | 0.843 | 0.13 | 0.495 | -0.06 | 0.770 |
| *PLCB3* | -0.01 | 0.939 | -0.17 | 0.378 | 0.35 | 0.063 |
| *PLCB4* | -0.18 | 0.354 | 0.33 | 0.077 | 0.14 | 0.461 |
| *PLCD1* | 0.01 | 0.953 | -0.13 | 0.489 | **0.37** | **0.045** |
| *PLCD3* | -0.20 | 0.291 | -0.18 | 0.352 | -0.15 | 0.449 |
| *PLCD4* | -0.03 | 0.858 | -0.15 | 0.443 | -0.19 | 0.317 |
| *PLCE1* | -0.20 | 0.307 | 0.01 | 0.960 | 0.07 | 0.725 |
| *PLCG1* | -0.16 | 0.405 | -0.04 | 0.838 | 0.08 | 0.683 |
| *PLCG2* | 0.13 | 0.487 | -0.08 | 0.667 | 0.21 | 0.281 |
| *PLCH1* | 0.05 | 0.815 | 0.22 | 0.260 | 0.23 | 0.232 |
| *PLCL1* | 0.03 | 0.880 | -0.21 | 0.275 | -0.06 | 0.744 |
| *PLCL2* | 0.27 | 0.163 | 0.16 | 0.407 | -0.01 | 0.950 |
| *PLD1* | -0.06 | 0.772 | 0.25 | 0.199 | 0.03 | 0.868 |
| *PLD2* | 0.19 | 0.329 | 0.09 | 0.659 | 0.35 | 0.065 |
| *PLD3* | -0.15 | 0.445 | -0.29 | 0.121 | **-0.42** | **0.024** |
| *PNPLA3* | -0.04 | 0.848 | 0.23 | 0.223 | 0.22 | 0.252 |
| *PNPLA6* | **0.41** | **0.028** | -0.11 | 0.576 | 0.13 | 0.501 |
| *PNPLA7* | 0.17 | 0.374 | 0.26 | 0.179 | **0.39** | **0.035** |
| *SLC22A1* | -0.03 | 0.897 | 0.08 | 0.689 | -0.09 | 0.659 |
| *SLC22A2* | -0.24 | 0.219 | 0.13 | 0.515 | -0.02 | 0.926 |
| *SLC44A1* | -0.14 | 0.454 | -0.20 | 0.286 | -0.10 | 0.622 |
| *SLC5A7* | 0.19 | 0.324 | 0.01 | 0.946 | 0.12 | 0.525 |

|  |  |  |  |  |  |  |
| --- | --- | --- | --- | --- | --- | --- |

Table 2:

| Gene | Cho |  | PCho |  | GPC |  |
| --- | --- | --- | --- | --- | --- | --- |
|  | ρ | p-value | ρ | p-value | ρ | p-value |
| *ASPG* | 0.20 | 0.409 | -0.21 | 0.379 | 0.02 | 0.950 |
| *CHKA* | **0.46** | **0.049** | **0.47** | **0.044** | **0.52** | **0.023** |
| *CHKB* | -0.16 | 0.519 | -0.08 | 0.756 | 0.14 | 0.566 |
| *CHPT1* | **0.50** | **0.029** | 0.16 | 0.511 | 0.35 | 0.142 |
| *CLC* | 0.15 | 0.539 | -0.03 | 0.912 | 0.03 | 0.904 |
| *GDPD1* | 0.09 | 0.719 | 0.02 | 0.945 | 0.05 | 0.834 |
| *GDPD2* | -0.35 | 0.143 | -0.20 | 0.420 | -0.30 | 0.211 |
| *GDPD3* | 0.17 | 0.487 | -0.32 | 0.188 | 0.17 | 0.496 |
| *GDPD4* | 0.45 | 0.051 | 0.35 | 0.139 | 0.38 | 0.112 |
| *GDPD5* | 0.37 | 0.115 | 0.41 | 0.080 | **0.73** | **<0.001** |
| *GPD1* | 0.34 | 0.152 | -0.07 | 0.775 | 0.11 | 0.647 |
| *GPD1L* | -0.04 | 0.882 | 0.13 | 0.591 | -0.07 | 0.790 |
| *GPD2* | 0.31 | 0.196 | -0.02 | 0.923 | -0.11 | 0.657 |
| *LCAT* | -0.30 | 0.207 | 0.03 | 0.914 | -0.16 | 0.521 |
| *LYPLA1* | -0.29 | 0.226 | -0.06 | 0.797 | -0.35 | 0.140 |
| *LYPLA2* | -0.03 | 0.907 | -0.36 | 0.131 | -0.08 | 0.758 |
| *PCYT1A* | 0.37 | 0.124 | -0.09 | 0.702 | 0.17 | 0.478 |
| *PCYT1B* | 0.03 | 0.895 | 0.10 | 0.680 | **0.49** | **0.035** |
| *PLA2G10* | **0.53** | **0.021** | 0.09 | 0.718 | 0.32 | 0.182 |
| *PLA2G12A* | -0.05 | 0.852 | -0.25 | 0.307 | -0.26 | 0.274 |
| *PLA2G12B* | -0.01 | 0.981 | -0.13 | 0.584 | 0.25 | 0.294 |
| *PLA2G15* | 0.08 | 0.745 | -0.27 | 0.270 | -0.02 | 0.947 |
| *PLA2G1B* | 0.39 | 0.100 | 0.12 | 0.625 | 0.02 | 0.924 |
| *PLA2G2A* | 0.30 | 0.217 | 0.08 | 0.737 | 0.04 | 0.859 |
| *PLA2G2D* | -0.36 | 0.133 | 0.15 | 0.547 | -0.19 | 0.426 |
| *PLA2G2E* | 0.17 | 0.484 | **-0.52** | **0.024** | -0.05 | 0.854 |
| *PLA2G2F* | -0.14 | 0.562 | -0.11 | 0.640 | 0.19 | 0.448 |
| *PLA2G3* | 0.10 | 0.673 | 0.33 | 0.163 | 0.24 | 0.316 |
| *PLA2G4A* | -0.05 | 0.851 | -0.31 | 0.191 | -0.27 | 0.270 |
| *PLA2G5* | 0.21 | 0.399 | 0.16 | 0.516 | 0.23 | 0.339 |
| *PLA2G6* | **0.61** | **0.006** | 0.32 | 0.177 | **0.67** | **0.002** |
| *PLCB1* | -0.21 | 0.399 | -0.06 | 0.801 | -0.33 | 0.172 |
| *PLCB2* | 0.32 | 0.185 | 0.33 | 0.164 | 0.41 | 0.080 |
| *PLCB3* | 0.08 | 0.747 | -0.12 | 0.622 | 0.33 | 0.167 |
| *PLCB4* | -0.09 | 0.704 | **0.51** | **0.027** | 0.18 | 0.449 |
| *PLCD1* | -0.05 | 0.841 | -0.20 | 0.407 | 0.20 | 0.417 |
| *PLCD3* | 0.11 | 0.657 | -0.17 | 0.480 | -0.01 | 0.973 |
| *PLCD4* | -0.21 | 0.383 | -0.18 | 0.451 | -0.28 | 0.245 |
| *PLCE1* | -0.29 | 0.229 | 0.14 | 0.565 | 0.10 | 0.680 |
| *PLCG1* | -0.30 | 0.205 | -0.03 | 0.905 | 0.01 | 0.968 |
| *PLCG2* | 0.07 | 0.785 | -0.18 | 0.469 | 0.20 | 0.419 |
| *PLCH1* | -0.23 | 0.346 | 0.11 | 0.653 | 0.15 | 0.549 |
| *PLCL1* | -0.23 | 0.340 | -0.29 | 0.227 | -0.26 | 0.284 |
| *PLCL2* | 0.24 | 0.324 | 0.21 | 0.390 | -0.03 | 0.909 |
| *PLD1* | -0.08 | 0.748 | 0.25 | 0.301 | 0.00 | 0.993 |
| *PLD2* | 0.26 | 0.281 | 0.03 | 0.893 | 0.27 | 0.259 |
| *PLD3* | -0.22 | 0.355 | -0.21 | 0.397 | -0.39 | 0.095 |
| *PNPLA3* | -0.05 | 0.846 | 0.33 | 0.171 | 0.13 | 0.608 |
| *PNPLA6* | 0.43 | 0.068 | -0.13 | 0.590 | 0.20 | 0.405 |
| *PNPLA7* | 0.38 | 0.109 | 0.41 | 0.078 | **0.52** | **0.021** |
| *SLC22A1* | 0.07 | 0.778 | -0.16 | 0.503 | -0.10 | 0.678 |
| *SLC22A2* | 0.02 | 0.946 | 0.35 | 0.140 | 0.09 | 0.714 |
| *SLC44A1* | -0.35 | 0.143 | -0.31 | 0.190 | -0.18 | 0.460 |
| *SLC5A7* | 0.18 | 0.454 | -0.36 | 0.135 | -0.24 | 0.332 |

Table 3:

| Gene | Cho |  | PCho |  | GPC |  |
| --- | --- | --- | --- | --- | --- | --- |
|  | ρ | p-value | ρ | p-value | ρ | p-value |
| *ASPG* | -0.65 | 0.160 | -0.47 | 0.348 | -0.69 | 0.131 |
| *CHKA* | -0.09 | 0.866 | 0.67 | 0.142 | 0.22 | 0.675 |
| *CHKB* | **-0.96** | **0.002** | -0.42 | 0.413 | **-0.97** | **0.001** |
| *CHPT1* | 0.60 | 0.210 | 0.01 | 0.988 | 0.34 | 0.515 |
| *CLC* | 0.12 | 0.828 | 0.15 | 0.775 | 0.20 | 0.704 |
| *GDPD1* | 0.13 | 0.804 | 0.11 | 0.841 | 0.28 | 0.585 |
| *GDPD2* | -0.09 | 0.867 | 0.24 | 0.646 | -0.01 | 0.982 |
| *GDPD3* | -0.38 | 0.463 | -0.75 | 0.084 | -0.54 | 0.272 |
| *GDPD4* | -0.35 | 0.503 | -0.47 | 0.352 | -0.60 | 0.210 |
| *GDPD5* | -0.40 | 0.437 | 0.02 | 0.974 | -0.23 | 0.658 |
| *GPD1* | -0.46 | 0.359 | -0.01 | 0.990 | -0.25 | 0.632 |
| *GPD1L* | -0.13 | 0.813 | -0.66 | 0.150 | -0.36 | 0.484 |
| *GPD2* | -0.26 | 0.613 | 0.32 | 0.539 | -0.03 | 0.961 |
| *LCAT* | -0.47 | 0.352 | -0.57 | 0.240 | -0.58 | 0.224 |
| *LYPLA1* | 0.47 | 0.349 | -0.57 | 0.234 | 0.07 | 0.901 |
| *LYPLA2* | -0.22 | 0.669 | -0.13 | 0.808 | -0.36 | 0.477 |
| *PCYT1A* | 0.07 | 0.902 | 0.51 | 0.299 | 0.23 | 0.659 |
| *PCYT1B* | 0.12 | 0.818 | 0.15 | 0.770 | -0.01 | 0.979 |
| *PLA2G10* | -0.12 | 0.822 | -0.32 | 0.534 | -0.31 | 0.553 |
| *PLA2G12A* | 0.22 | 0.680 | -0.24 | 0.640 | 0.06 | 0.913 |
| *PLA2G12B* | **-0.91** | **0.012** | -0.02 | 0.975 | -0.74 | 0.092 |
| *PLA2G15* | -0.72 | 0.108 | 0.01 | 0.985 | -0.49 | 0.324 |
| *PLA2G1B* | 0.50 | 0.313 | -0.28 | 0.593 | 0.40 | 0.435 |
| *PLA2G2A* | 0.18 | 0.738 | 0.11 | 0.833 | 0.23 | 0.660 |
| *PLA2G2D* | -0.28 | 0.595 | -0.22 | 0.669 | -0.33 | 0.518 |
| *PLA2G2E* | 0.40 | 0.426 | 0.64 | 0.171 | 0.56 | 0.250 |
| *PLA2G2F* | 0.33 | 0.525 | 0.61 | 0.195 | 0.50 | 0.316 |
| *PLA2G3* | -0.17 | 0.751 | -0.70 | 0.119 | -0.26 | 0.625 |
| *PLA2G4A* | **0.86** | **0.029** | -0.17 | 0.747 | 0.71 | 0.114 |
| *PLA2G5* | -0.32 | 0.536 | 0.46 | 0.360 | -0.22 | 0.670 |
| *PLA2G6* | 0.05 | 0.918 | -0.15 | 0.771 | -0.03 | 0.957 |
| *PLCB1* | -0.22 | 0.672 | **-0.91** | **0.011** | -0.48 | 0.341 |
| *PLCB2* | -0.76 | 0.080 | 0.06 | 0.909 | -0.66 | 0.153 |
| *PLCB3* | 0.22 | 0.679 | -0.21 | 0.694 | 0.00 | 0.996 |
| *PLCB4* | -0.55 | 0.259 | -0.17 | 0.741 | -0.62 | 0.192 |
| *PLCD1* | -0.42 | 0.404 | -0.35 | 0.499 | -0.38 | 0.459 |
| *PLCD3* | **-0.86** | **0.027** | -0.47 | 0.348 | **-0.81** | **0.049** |
| *PLCD4* | 0.36 | 0.481 | -0.49 | 0.329 | -0.05 | 0.929 |
| *PLCE1* | -0.11 | 0.835 | 0.01 | 0.986 | -0.16 | 0.755 |
| *PLCG1* | 0.50 | 0.314 | -0.10 | 0.854 | 0.34 | 0.507 |
| *PLCG2* | -0.13 | 0.799 | -0.09 | 0.859 | 0.01 | 0.982 |
| *PLCH1* | 0.54 | 0.266 | 0.35 | 0.502 | 0.70 | 0.123 |
| *PLCL1* | 0.72 | 0.106 | -0.24 | 0.652 | 0.46 | 0.363 |
| *PLCL2* | 0.72 | 0.107 | 0.11 | 0.840 | 0.61 | 0.195 |
| *PLD1* | 0.10 | 0.844 | **0.89** | **0.017** | 0.48 | 0.332 |
| *PLD2* | -0.37 | 0.476 | -0.09 | 0.870 | -0.36 | 0.479 |
| *PLD3* | 0.13 | 0.809 | -0.61 | 0.194 | -0.08 | 0.878 |
| *PNPLA3* | -0.05 | 0.931 | 0.74 | 0.095 | 0.24 | 0.647 |
| *PNPLA6* | 0.42 | 0.408 | 0.09 | 0.863 | 0.41 | 0.416 |
| *PNPLA7* | 0.30 | 0.569 | 0.55 | 0.260 | 0.64 | 0.171 |
| *SLC22A1* | -0.05 | 0.923 | 0.80 | 0.054 | 0.26 | 0.619 |
| *SLC22A2* | -0.79 | 0.064 | -0.49 | 0.322 | **-0.86** | **0.030** |
| *SLC44A1* | 0.03 | 0.953 | -0.41 | 0.419 | -0.32 | 0.531 |
| *SLC5A7* | -0.28 | 0.587 | 0.34 | 0.516 | -0.19 | 0.719 |
