# Supplementary material for: Interplay of choline metabolites and genes in patient-derived breast cancer xenografts
Source: Breast Cancer Res. 2014 Jan 21;16(1):R5. doi: 10.1186/bcr3597 (PMC3978476; doi:10.1186/bcr3597)
Supplement: Additional file 3: Table S4 — Mean choline gene expressions for basal-like and luminal B, difference in expression basal-like-luminal B, P-values and FDR values, for patient tissue samples and xenografts. All genes being significantly differently expressed for basal-like – luminal B (fdr <0.1) are emphasized in bold. [file bcr3597-S3.doc]

# Additional file 3:

|  | **Patient Cohort 3** | | | | | **Xenografts** | | | | |
| --- | --- | --- | --- | --- | --- | --- | --- | --- | --- | --- |
|  | **Basal** | **Luminal B** |  |  |  | **Basal** | **Luminal B** |  |  |  |
| **Gene** | **mean** | **mean** | **Difference** | **p-value** | **fdr** | **mean** | **mean** | **Difference** | **p-value** | **fdr** |
| *ASPG* | - | - | - | - | - | 5.87 | 6.01 | -0.14 | 0.722 | 0.875 |
| *CHKA* | **10.20** | **9.54** | **0.66** | **0.007** | **0.039** | 11.53 | 10.96 | 0.57 | 0.097 | 0.261 |
| *CHKB* | 11.53 | 11.74 | -0.21 | 0.360 | 0.495 | 9.19 | 8.33 | 0.86 | 0.034 | 0.139 |
| *CHPT1* | 10.36 | 9.88 | 0.48 | 0.231 | 0.416 | 9.39 | 8.77 | 0.62 | 0.142 | 0.328 |
| *CLC* | 6.47 | 6.37 | 0.10 | 0.438 | 0.576 | 4.99 | 5.06 | -0.06 | 0.022 | 0.120 |
| *GDPD1* | **6.33** | **6.58** | **-0.25** | **0.034** | **0.098** | 5.60 | 6.10 | -0.50 | 0.102 | 0.263 |
| *GDPD2* | 6.69 | 6.97 | -0.28 | 0.254 | 0.429 | 5.25 | 5.54 | -0.29 | 0.391 | 0.621 |
| *GDPD3* | **7.42** | **8.20** | **-0.78** | **0.032** | **0.098** | **6.26** | **8.07** | **-1.81** | **4.E-04** | **0.006** |
| *GDPD4* | 6.15 | 6.18 | -0.03 | 0.622 | 0.764 | 5.13 | 5.17 | -0.04 | 0.519 | 0.749 |
| *GDPD5* | **8.97** | **8.23** | **0.74** | **0.024** | **0.094** | 6.61 | 6.63 | -0.02 | 0.953 | 0.990 |
| *GPD1* | 7.82 | 7.95 | -0.14 | 0.707 | 0.848 | 6.08 | 5.92 | 0.16 | 0.541 | 0.749 |
| *GPD1L* | **8.05** | **8.84** | **-0.79** | **3.E-04** | **0.003** | **8.17** | **10.61** | **-2.45** | **4.E-06** | **2.E-04** |
| *GPD2* | 8.26 | 8.25 | 0.01 | 0.939 | 0.971 | 8.78 | 9.40 | -0.63 | 0.056 | 0.182 |
| *LCAT* | **7.62** | **7.18** | **0.43** | **0.002** | **0.014** | **7.42** | **6.32** | **1.10** | **0.006** | **0.057** |
| *LYPLA1* | **10.17** | **10.71** | **-0.53** | **0.033** | **0.098** | 10.08 | 10.84 | -0.76 | 0.028 | 0.138 |
| *LYPLA2* | 7.59 | 7.73 | -0.14 | 0.323 | 0.474 | 9.77 | 9.77 | -0.01 | 0.975 | 0.991 |
| *PCYT1A* | 8.45 | 8.49 | -0.03 | 0.870 | 0.940 | 8.40 | 8.76 | -0.37 | 0.201 | 0.391 |
| *PCYT1B* | **6.50** | **6.27** | **0.23** | **0.033** | **0.098** | 5.47 | 5.11 | 0.35 | 0.057 | 0.182 |
| *PLA2G10* | **6.75** | **8.27** | **-1.52** | **1.E-05** | **0.001** | 6.06 | 6.83 | -0.77 | 0.085 | 0.241 |
| *PLA2G12A* | **9.63** | **10.09** | **-0.46** | **0.033** | **0.098** | **9.06** | **9.81** | **-0.75** | **0.008** | **0.057** |
| *PLA2G12B* | 6.09 | 6.15 | -0.07 | 0.216 | 0.411 | 4.71 | 4.72 | 0.00 | 0.943 | 0.990 |
| *PLA2G15* | 9.25 | 9.49 | -0.24 | 0.221 | 0.411 | 11.21 | 11.21 | 0.00 | 0.991 | 0.991 |
| *PLA2G1B* | 6.45 | 6.44 | 0.00 | 0.957 | 0.971 | 5.61 | 5.43 | 0.18 | 0.203 | 0.391 |
| *PLA2G2A* | 7.66 | 7.03 | 0.63 | 0.127 | 0.280 | 5.10 | 5.15 | -0.06 | 0.152 | 0.328 |
| *PLA2G2D* | 6.79 | 6.89 | -0.10 | 0.325 | 0.474 | 5.26 | 5.23 | 0.02 | 0.706 | 0.875 |
| *PLA2G2E* | 6.15 | 6.15 | -0.01 | 0.776 | 0.911 | 4.72 | 4.75 | -0.04 | 0.460 | 0.710 |
| *PLA2G2F* | 6.32 | 6.41 | -0.09 | 0.072 | 0.177 | 5.32 | 5.56 | -0.24 | 0.047 | 0.169 |
| *PLA2G3* | 6.82 | 6.68 | 0.14 | 0.449 | 0.577 | 5.21 | 5.34 | -0.13 | 0.527 | 0.749 |
| *PLA2G4A* | **8.54** | **7.39** | **1.15** | **3.E-04** | **0.003** | 6.74 | 5.11 | 1.63 | 0.035 | 0.139 |
| *PLA2G4B* | 11.06 | 11.27 | -0.20 | 0.367 | 0.495 | - | - | - | - | - |
| *PLA2G5* | 7.37 | 7.34 | 0.03 | 0.864 | 0.940 | 5.66 | 5.70 | -0.04 | 0.372 | 0.609 |
| *PLA2G6* | **7.88** | **8.32** | **-0.44** | **0.010** | **0.054** | 6.83 | 6.69 | 0.13 | 0.280 | 0.487 |
| *PLCB1* | 8.23 | 8.15 | 0.08 | 0.803 | 0.923 | 7.98 | 8.14 | -0.16 | 0.803 | 0.903 |
| *PLCB2* | 7.09 | 6.87 | 0.21 | 0.040 | 0.108 | 8.37 | 8.41 | -0.04 | 0.933 | 0.990 |
| *PLCB3* | 8.06 | 7.84 | 0.21 | 0.247 | 0.429 | 6.64 | 6.52 | 0.12 | 0.508 | 0.749 |
| *PLCB4* | 8.20 | 7.96 | 0.24 | 0.356 | 0.495 | 7.10 | 7.21 | -0.11 | 0.737 | 0.875 |
| *PLCD1* | **10.04** | **9.50** | **0.54** | **0.024** | **0.094** | **9.64** | **8.34** | **1.29** | **0.008** | **0.057** |
| *PLCD3* | 6.86 | 6.68 | 0.18 | 0.121 | 0.280 | 5.68 | 6.03 | -0.35 | 0.133 | 0.326 |
| *PLCD4* | **6.57** | **7.69** | **-1.12** | **3.E-05** | **0.001** | **5.90** | **8.01** | **-2.11** | **4.E-04** | **0.006** |
| *PLCE1* | **9.29** | **7.94** | **1.35** | **1.E-04** | **0.002** | **8.02** | **6.64** | **1.38** | **0.012** | **0.073** |
| *PLCG1* | 8.48 | 8.62 | -0.14 | 0.289 | 0.471 | 8.01 | 8.11 | -0.10 | 0.592 | 0.780 |
| *PLCG2* | **10.79** | **9.79** | **1.00** | **0.004** | **0.028** | **8.05** | **5.42** | **2.62** | **1.E-04** | **0.003** |
| *PLCH1* | **7.09** | **6.71** | **0.38** | **0.017** | **0.084** | 6.61 | 6.27 | 0.34 | 0.340 | 0.573 |
| *PLCL1* | 6.67 | 6.68 | -0.01 | 0.957 | 0.971 | 5.85 | 5.30 | 0.55 | 0.175 | 0.364 |
| *PLCL2* | 7.58 | 7.37 | 0.22 | 0.171 | 0.356 | 5.42 | 5.16 | 0.26 | 0.269 | 0.485 |
| *PLD1* | **7.65** | **7.16** | **0.49** | **2.E-04** | **0.003** | 6.68 | 5.81 | 0.86 | 0.065 | 0.195 |
| *PLD2* | 7.30 | 7.12 | 0.18 | 0.130 | 0.280 | 11.19 | 10.77 | 0.41 | 0.151 | 0.328 |
| *PLD3* | 8.10 | 8.07 | 0.03 | 0.850 | 0.940 | 9.31 | 9.44 | -0.13 | 0.654 | 0.841 |
| *PNPLA3* | 8.50 | 7.71 | 0.79 | 0.042 | 0.109 | **10.00** | **8.32** | **1.69** | **0.008** | **0.057** |
| *PNPLA6* | 8.14 | 8.14 | 0.00 | 0.971 | 0.971 | 7.41 | 7.24 | 0.17 | 0.582 | 0.780 |
| *PNPLA7* | 7.16 | 7.33 | -0.17 | 0.296 | 0.471 | 7.98 | 8.77 | -0.79 | 0.036 | 0.139 |
| *SLC22A1* | 6.37 | 6.28 | 0.09 | 0.200 | 0.399 | 5.75 | 5.79 | -0.04 | 0.745 | 0.875 |
| *SLC22A2* | 6.49 | 6.46 | 0.03 | 0.465 | 0.584 | 5.10 | 5.11 | -0.01 | 0.827 | 0.911 |
| *SLC44A1* | 11.02 | 11.18 | -0.15 | 0.314 | 0.474 | 10.03 | 9.95 | 0.08 | 0.784 | 0.901 |
| *SLC5A7* | **6.19** | **6.39** | **-0.20** | **0.023** | **0.094** | 5.17 | 5.23 | -0.06 | 0.253 | 0.471 |
